# Clinical Outcomes of *Candida auris* Versus Other *Candida* Species Bloodstream Infections: An IPTW-Adjusted Cohort Study in South Korea

**DOI:** 10.3390/jof12070495

**Published:** 2026-07-06

**Authors:** Mindong Sung, Shihwan Jang, Ah Young Leem, Chan Ho Lee, Kyung Soo Chung, Young Sam Kim, Joon Kim, Su Hwan Lee

**Affiliations:** 1 Division of Pulmonary and Critical Care Medicine, Department of Internal Medicine, Severance Hospital, Yonsei University College of Medicine, Seoul 03722, Republic of Korea; mdsung@yuhs.ac (M.S.);; 2 Institute for Innovation in Digital Healthcare, Severance Hospital, Yonsei University College of Medicine, Seoul 03722, Republic of Korea; 3 Division of Pulmonology, Department of Internal Medicine, Inha University Hospital, Inha University College of Medicine, Incheon 22332, Republic of Korea

**Keywords:** *Candida auris*, candidaemia, bloodstream infection, antifungal susceptibility, mortality, inverse probability of treatment weighting, East Asia

## Abstract

*Candida auris* has emerged as a multidrug-resistant, healthcare-associated pathogen worldwide; however, outcome data on *C. auris* candidaemia in East Asia remain limited. We conducted a retrospective cohort study of adult patients with candidaemia who received antifungal therapy at a tertiary hospital in Seoul, Republic of Korea, from January 2023 to December 2024, comparing *C. auris* with other *Candida* species. Confounding was addressed by inverse probability of treatment weighting (IPTW) using a five-covariate propensity score (age, Charlson Comorbidity Index, septic shock, ICU admission at antifungal initiation, and concomitant Gram-negative infection). Among 423 patients, *C. auris* accounted for 6.9% of cases and was uniformly fluconazole non-susceptible, with frequent high-level caspofungin resistance but preserved micafungin and anidulafungin susceptibility. Patients with *C. auris* were older, with greater comorbidity and more frequent ICU admission at antifungal initiation. After IPTW adjustment, *C. auris* was not associated with higher 30-day mortality, the primary outcome (adjusted hazard ratio 0.59, 95% CI 0.26–1.32); the wide confidence interval indicates limited precision rather than equivalence, and results were directionally consistent for 90-day and in-hospital mortality and across sensitivity analyses that varied both the comparison cohort and the analytic method. Residual confounding by unmeasured illness severity and limited precision preclude concluding equivalence. Continued surveillance, molecular characterisation, and infection control remain essential.

## 1. Introduction

*Candida* species are a major cause of healthcare-associated bloodstream infections (BSIs) and are consistently ranked among the leading pathogens in hospitalised patients [1,2,3]. In the United States, *Candida* spp. represent the fourth most common cause of nosocomial BSI, accounting for approximately 8–10% of all hospital-acquired BSIs, with crude mortality rates approaching 40% [4]. The disease mainly affects individuals with extensive healthcare exposure, such as those with central venous catheters, broad-spectrum antibiotic use, total parenteral nutrition, recent surgery, or immunosuppression [3]. Delays in diagnosis and initiation of appropriate antifungal treatment have consistently been associated with increased mortality [5,6].

*Candida auris*, first described in Japan in 2009, has emerged as a globally disseminated nosocomial pathogen [7,8]. Surveillance studies indicate that *C. auris* currently accounts for approximately 0.4–1.6% of candidaemia cases in global surveillance programmes [9], with increasing reports of hospital outbreaks and inter-facility transmission [10]. Owing to its rapid spread and multidrug resistance, the World Health Organization has designated *C. auris* as a critical-priority fungal pathogen [11]. Nevertheless, several epidemiological studies suggest that the mortality of *C. auris* candidaemia is broadly comparable to that of other non-albicans *Candida* species [12,13].

Experimental studies indicate that *C. auris* possesses distinct biological characteristics that differentiate it from other *Candida* species. In vitro and in vivo investigations indicate that *C. auris* exhibits high antifungal tolerance, frequent multidrug resistance, robust biofilm formation, prolonged environmental persistence on hospital surfaces, and enhanced skin colonisation [14,15,16,17]. These features facilitate healthcare-associated transmission and outbreak potential. However, in several infection models, the intrinsic virulence of *C. auris* has been comparable to or lower than that of *C. albicans*, suggesting that its clinical impact may be driven more by persistence and transmissibility than by increased pathogenicity [18,19].

Despite the increasing global recognition of *C. auris*, epidemiologic and outcome data from East Asia—and from the Republic of Korea specifically—remain scarce. Earlier surveillance from the Republic of Korea characterising the species distribution and azole susceptibility of candidaemia isolates predated the emergence of *C. auris* [20]. More recent reports from this region are limited to sporadic case reports, isolate-based laboratory studies, and infection-control surveillance, with few data addressing the clinical epidemiology or outcomes of *C. auris* candidaemia [21,22]. Notably, regional clade distribution and antifungal susceptibility patterns appear to differ from those reported in North American or European cohorts, underscoring the need for direct East Asian outcome data [23]. In this study, we investigated the epidemiology, antifungal susceptibility, clinical characteristics, and outcomes of candidaemia—with a particular focus on *C. auris*—at a tertiary referral hospital in the Republic of Korea, using inverse probability of treatment weighting to adjust for confounding.

## 2. Materials and Methods

### 2.1. Study Design and Patient Population

We retrospectively reviewed the electronic medical records of patients with *Candida* bloodstream infection (BSI) at Yonsei University Severance Hospital, a 2111-bed tertiary referral centre in Seoul, Republic of Korea, from January 2023 to December 2024. Adult patients (aged ≥ 18 years) who received any systemic antifungal therapy for candidaemia—and were therefore alive at the initiation of treatment—were eligible. Patients younger than 18 years and those with no documented antifungal treatment were excluded. This relaxed inclusion defines a clinically representative cohort of treated candidaemia (the main cohort); a subset treated with a first-line echinocandin (the echinocandin-restricted cohort) is additionally defined for the analyses below.

Patients were followed for up to 1 year after the candidaemia diagnosis. Observation beyond 1 year was censored at 1 year; patients who were lost to follow-up were censored at the last clinical contact.

### 2.2. Target Trial Framework and Time Zero

We framed the analysis as a target-trial emulation in which time zero was the first positive blood culture for *Candida* (the index date) and the contrast of interest was *C. auris* versus other *Candida* species. Because eligibility required survival to, and receipt of, antifungal therapy, the cohort is a treated-survivor population; the quantity estimated is therefore 30-day mortality conditional on being alive and treated at time zero, rather than the effect of species among all incident candidaemia. This design can induce selection or immortal-time bias if the interval from culture to treatment, or early-death risk, differs by species. We addressed this by indexing survival time to the culture date and by a landmark sensitivity analysis restricted to patients alive at 48 h (Statistical analysis), and we describe between-species differences in treatment timing as a limitation.

### 2.3. Data Collection

Clinical, microbiological, and treatment-related data were retrospectively extracted from electronic medical records, comprising demographic characteristics, comorbidities, *Candida* species identification and the antifungal susceptibility category of the index blood isolate, antifungal treatment details, prior healthcare exposure, and clinical outcomes.

### 2.4. Study Definitions

Candidaemia was defined as at least one blood culture positive for a *Candida* species. Comorbidity burden was summarised using the Charlson Comorbidity Index (CCI), weighted per the original Charlson scheme [24]. Acute illness severity was captured by two objectively ascertainable indicators available for all patients—septic shock and intensive care unit (ICU) admission at the time of antifungal initiation.

Septic shock was defined according to the Sepsis-3 criteria [25] as persistent hypotension requiring vasopressor support to maintain a mean arterial pressure ≥65 mmHg with a serum lactate level >2 mmol/L. Concomitant Gram-negative infection was defined as the presence of any of three site-specific findings within 14 days before or after the index blood culture: Gram-negative bacteraemia, Gram-negative pneumonia (a Gram-negative pathogen from a respiratory specimen with concurrent clinical or radiologic pneumonia), or Gram-negative urinary tract infection (a Gram-negative pathogen from urine with concurrent clinical documentation). Concurrent multidrug-resistant bacterial co-infection (extended-spectrum β-lactamase-producing Enterobacterales, methicillin-resistant *Staphylococcus aureus*, vancomycin-resistant *Enterococcus*, multidrug-resistant *Pseudomonas aeruginosa*, multidrug-resistant *Acinetobacter baumannii*, and carbapenem-resistant Enterobacterales) was defined as recovery of one of these organisms from a blood culture or from a respiratory specimen with concurrent clinical pneumonia within 7 days of the index candidaemia. These data were derived objectively from microbiology and antimicrobial-susceptibility records for the full cohort. This concurrent co-infection is distinct from prior multidrug-resistant colonisation within 180 days (Table 1) and from surveillance stool cultures, which were not counted.

The presumed source of candidaemia was adjudicated from the electronic medical record for the full cohort. A urinary source required isolation of the same *Candida* species from a urine culture obtained within 48 h of the index blood culture together with clinical or radiologic features of urinary tract infection, whereas sustained candiduria in the absence of urinary symptoms or radiologic findings was not classified as a urinary source; an intravascular-device source required isolation of the same species from a catheter-tip culture (catheter-related candidaemia), and an intra-abdominal source required isolation of the same species from an intra-abdominal specimen with a compatible clinical or radiologic focus. Source was recorded descriptively and was not used in the adjusted analysis.

Antifungal susceptibility testing (AFST) was not performed routinely on every isolate at our institution; testing was requested at the discretion of the treating clinician and the microbiology laboratory, generally when additional susceptibility information was considered clinically relevant for patient management, and the susceptibility categories analysed here are those recorded in the electronic medical record.

### 2.5. Outcomes

The primary outcome was 30-day all-cause mortality. Secondary outcomes were 90-day all-cause mortality, in-hospital all-cause mortality, and day-14 all-cause mortality. Pre-specified exploratory analyses comprised microbiological clearance (documented conversion of follow-up blood cultures to negative, ascertainable only in patients with documented follow-up cultures), the interval from candidaemia onset to death, the antifungal susceptibility profile of the index isolates, and—within the echinocandin-restricted cohort—the baseline hepatic profile by first-line agent.

### 2.6. Statistical Analysis

Continuous variables were compared using the Mann–Whitney U test and categorical variables using Fisher’s exact test. To adjust for confounding, we applied inverse probability of treatment weighting (IPTW). Propensity scores were estimated by multivariable logistic regression on five pre-specified covariates: age, CCI, septic shock, ICU admission at antifungal initiation, and concomitant Gram-negative infection. The presumed candidaemia source was deliberately excluded from the propensity-score model because it is a descriptive characteristic of the infection episode that may lie on the causal pathway between species and outcome, rather than a stable pre-treatment confounder. From the propensity scores we derived stabilised weights, trimmed at the 1st and 99th percentiles. Covariate balance was assessed using absolute standardised mean differences (SMDs), with values < 0.1 considered adequate, and visualised in a Love plot.

For the primary outcome (30-day all-cause mortality), we estimated an adjusted hazard ratio (aHR) from a weighted Cox proportional hazards model and report it alongside a doubly robust estimate combining IPTW with outcome-model covariate adjustment. The 90-day outcome was analysed by weighted Cox regression (aHR). In-hospital mortality, being horizon-free, was analysed by weighted logistic regression (adjusted odds ratio); its odds ratio is not directly comparable to the time-to-event hazard ratios. Day-14 mortality was analysed by weighted logistic regression (adjusted odds ratio), whereas microbiological clearance, the onset-to-death interval, and the antifungal susceptibility profile (the proportion of isolates susceptible per species) were summarised descriptively. The proportional hazards assumption was evaluated using Schoenfeld residuals. Because the 30- and 90-day outcomes were modelled as time-to-event endpoints, patients with incomplete follow-up were right-censored at their last clinical contact; the hazard ratios and Kaplan–Meier estimates therefore reflect the at-risk population over time rather than a fixed denominator, so that limited follow-up reduces precision at later horizons without biassing the mortality estimate.

As an exploratory analysis restricted to the echinocandin-restricted cohort, baseline hepatic profile (serum bilirubin and clinically recognised liver disease) was compared across first-line echinocandin agents using the Mann–Whitney U test and Fisher’s exact test, to examine determinants of agent selection.

We pre-specified two layers of sensitivity analysis for the primary outcome. The first varied the patient set under the same five-covariate model: the echinocandin-treated cohort, a bacteraemia-excluded cohort (any positive non-*Candida* blood culture within 14 days removed), and comparator-restricted analyses against *C. albicans* alone and against non-albicans *Candida* species. The second applied alternative estimators to the main cohort: overlap weights (targeting the equipoise population), a Firth penalised propensity score, a landmark analysis restricted to patients alive at 48 h, and a Fine–Gray competing-risk model for in-hospital mortality treating discharge alive as the competing event. Propensity-score diagnostics were score overlap, the range of stabilised weights, and a leave-one-out influence check omitting each *C. auris* case in turn.

All statistical tests were two-sided, with *p* < 0.05 considered significant. Analyses were performed using R version 4.3.3 (R Foundation for Statistical Computing, Vienna, Austria).

### 2.7. Ethical Review

This study was approved by the Institutional Review Board of Severance Hospital (IRB No. 4-2025-0375). The requirement for informed consent was waived owing to the retrospective nature of the study.

## 3. Results

### 3.1. Patient Eligibility and Monthly Incidence of Candidaemia

**Figure 1:**
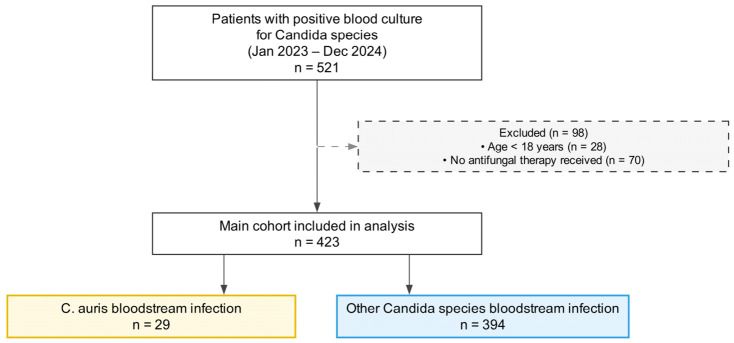
Flowchart of patient eligibility assessment. Screening, exclusions, and the resulting main cohort over the study period (January 2023 to December 2024). BSI, bloodstream infection.

From January 2023 to December 2024, 521 patients had at least one blood culture positive for *Candida* species and were screened for eligibility. After excluding 28 patients younger than 18 years and 70 who received no antifungal therapy, 423 patients were included, comprising 29 cases of *C. auris* bloodstream infection (BSI) and 394 cases of BSI caused by other *Candida* species (Figure 1). Eligibility required only receipt of systemic antifungal therapy rather than a specific first-line agent; these 423 treated patients constituted the analytic cohort and served as the denominator for all subsequent analyses.

**Figure 2:**
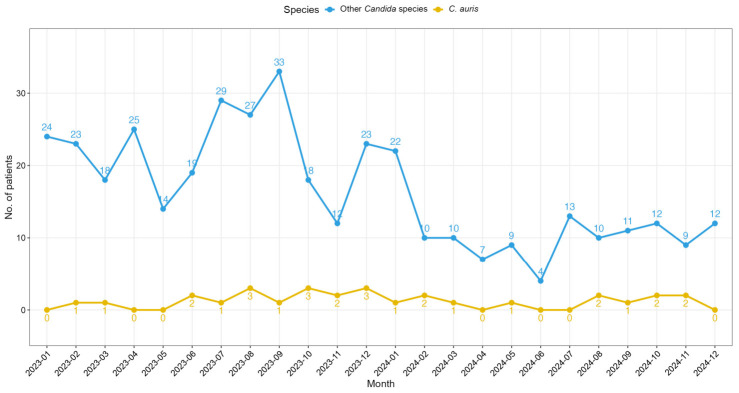
Monthly incidence of candidaemia by species. Number of patients diagnosed with candidaemia per month at a tertiary referral hospital from January 2023 to December 2024, stratified by *Candida auris* and other *Candida* species.

*C. auris* BSI occurred at a mean of approximately 1.2 cases per month and BSI caused by other *Candida* species at approximately 16.4 cases per month. The monthly distribution by species is shown in Figure 2 and Supplementary Table S1.

### 3.2. Patient Demographics and Healthcare Exposure Characteristics

Baseline demographics are summarised in Table 1. Patients with *C. auris* BSI were older than those with candidaemia caused by other *Candida* species (median 72 vs. 67 years, *p* = 0.012) and carried a higher comorbidity burden (Charlson Comorbidity Index 8 vs. 6, *p* < 0.001). They were also more frequently in the intensive care unit at the time of antifungal initiation and had a longer in-hospital interval from admission to the onset of candidaemia. Other demographic, healthcare-exposure, and host factors—including sex, body mass index, recent hospitalisation, prior antifungal exposure, surgery, multidrug-resistant organism colonisation, neutropenia, and septic shock at onset—did not differ significantly between groups. Kidney transplantation, a host factor not captured by the index, was uncommon in both groups. The full panel of individual comorbidities is provided in Supplementary Table S2.

### 3.3. Clinical and Infection Characteristics of the Patients

The clinical and infection-related characteristics are summarised in Table 2. The presumed source of candidaemia was an intravascular device in 49% of patients overall and was not identifiable in 34%, with a urinary source in 15% and an intra-abdominal source in 2%. Concomitant Gram-negative infection was numerically more frequent in the *C. auris* group, but the source distribution, the burden of concomitant Gram-negative, any bacterial, and concurrent multidrug-resistant co-infection, the distribution of first-line antifungal agents, the total duration of antifungal therapy, and the proportion with documented microbiological clearance did not differ significantly between groups (Table 2). Fluconazole was the most common first-line agent overall (46%), and the per-pathogen multidrug-resistant distribution is provided in Supplementary Table S3.

### 3.4. Covariate Balance After IPTW

**Figure 3:**
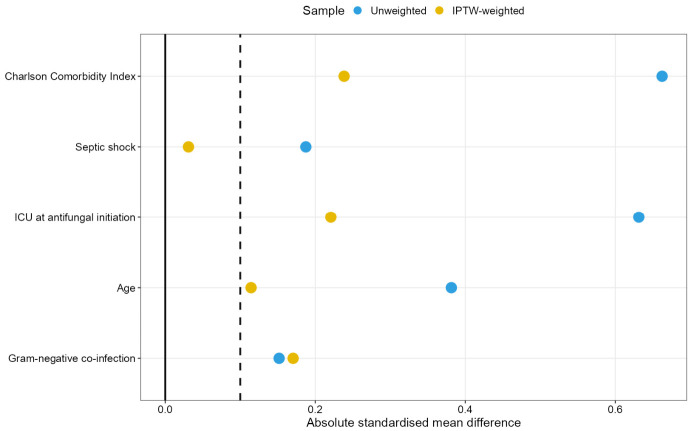
Love plot of standardised mean differences before and after IPTW adjustment. Absolute standardised mean differences for the five propensity-score covariates in the unweighted sample and after inverse probability of treatment weighting. The dashed vertical line indicates the balance threshold of 0.1; residual standardised mean differences after weighting are reported in Section 3.

After applying stabilised, trimmed inverse probability weights, the absolute standardised mean difference (SMD) for age fell to 0.11 and that for septic shock to 0.03 (Figure 3). Residual imbalance remained, however, for the Charlson Comorbidity Index (SMD 0.24), ICU status at antifungal initiation (0.22), and Gram-negative co-infection (0.17), reflecting the limited overlap of propensity scores between *C. auris* (n = 29; effective sample size after weighting, 20.9) and comparator patients (Supplementary Figure S1). Doubly robust analyses, reported below, were specified a priori to reduce reliance on perfect weighting balance.

### 3.5. Patient Clinical Outcomes

The median follow-up was 18 days (interquartile range [IQR], 9–42) overall, 22 days (IQR 12–64) in the *C. auris* group, and 18 days (IQR 9–40) in the comparator group. Only 7 of 29 *C. auris* patients (24%) and 49 of 394 comparator patients (12%) were under observation through the full 90-day window, limiting information on late post-acute outcomes.

**Figure 4:**
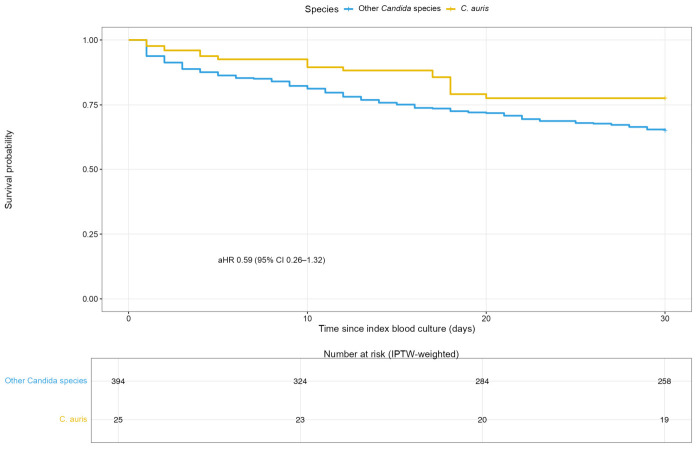
Inverse probability of treatment weighting (IPTW)-adjusted Kaplan–Meier survival curves based on *Candida* species. Inverse probability of treatment weighted 30-day Kaplan–Meier survival curves for patients with *C. auris* BSI (n = 29) versus candidaemia caused by other *Candida* species (n = 394), with the integrated number-at-risk table. The annotation displays the adjusted hazard ratio for 30-day mortality (the primary outcome) from the IPTW-weighted Cox proportional hazards model. The number-at-risk counts are IPTW-weighted and therefore differ from the unweighted patient counts. The unadjusted Kaplan–Meier curves are presented in Supplementary Figure S2.

Clinical outcomes are summarised in Table 3. In the unadjusted analyses, mortality was similar between groups. After IPTW, *C. auris* BSI was not associated with increased 30-day mortality, the primary outcome (adjusted hazard ratio [aHR], 0.59; 95% CI, 0.26–1.32), nor with 90-day mortality (aHR, 0.76; 95% CI, 0.43–1.35) or in-hospital mortality (adjusted odds ratio [aOR], 1.01; 95% CI, 0.42–2.45); the proportional-hazards assumption was not violated for the time-to-event models (Schoenfeld residuals, *p* > 0.05). Doubly robust models incorporating both IPTW and outcome-model covariate adjustment yielded a consistent primary estimate (aHR, 0.64; 95% CI, 0.28–1.49). The IPTW-adjusted Kaplan–Meier survival curves over the 30-day follow-up are presented in Figure 4, with IPTW-weighted numbers at risk indicated.

Day-14 mortality was 6 of 29 (21%) in the *C. auris* group versus 96 of 394 (24%) in the comparator group (IPTW-weighted aOR, 0.42; 95% CI, 0.15–1.13). Time to microbiological clearance, assessable only in the subset with serial follow-up cultures (n = 237), was similar between groups (median approximately 4 days).

Among decedents, *C. auris* deaths occurred later than comparator deaths (43% vs. 54% within 14 days of onset; 36% vs. 22% beyond day 30; median onset-to-death, 17.5 vs. 13 days).

### 3.6. Exploratory Analyses

Antifungal susceptibility profiles: AFST results on the index blood isolate were available for 15 of 29 *C. auris* patients (52%) and for 216 of 394 patients (55%) with other *Candida* species (per-drug tested and untested counts in Supplementary Table S4). Among tested *C. auris* isolates, 14 of 15 (93%) were fluconazole-resistant and 13 of 15 (87%) caspofungin-resistant, whereas micafungin and anidulafungin susceptibility was preserved (13/15 [87%] and 12/13 [92%] susceptible, respectively). In the comparator group, caspofungin non-susceptibility was concentrated almost entirely in *C. glabrata*; overall, 36 of 38 caspofungin non-susceptible isolates (95%) were *C. auris* or *C. glabrata*.

Baseline hepatic profile by first-line antifungal agent: Within the echinocandin-restricted cohort (n = 237; the predefined sensitivity cohort), baseline hepatic profile differed by first-line agent. Patients receiving anidulafungin (n = 53) had a higher baseline serum bilirubin (median 3.05 mg/dL, IQR 1.08–7.15) than patients receiving caspofungin (n = 161; 0.90 mg/dL, IQR 0.60–1.80) or micafungin (n = 23; 1.25 mg/dL, IQR 1.02–2.15), and a higher prevalence of clinically recognised liver disease (37.7% vs. 5.6% and 17.4%, respectively).

### 3.7. Sensitivity Analyses

The sensitivity analyses are summarised in Table 4. Across all alternative cohort definitions—restriction to first-line echinocandin recipients (the predefined sensitivity cohort; aHR, 0.42), exclusion of concomitant bacteraemia, and comparator restriction to *C. albicans* alone or to non-albicans *Candida* species—and across all alternative estimators—doubly robust and overlap weighting, a 48 h landmark analysis, a Firth-penalised model, and a horizon-free Fine–Gray competing-risk model—the point estimates remained at or below unity, with every confidence interval spanning unity (aHR range, 0.42–0.92; Table 4). Adding the presumed candidaemia source as a sixth propensity-score covariate left the estimate essentially unchanged.

## 4. Discussion

In this retrospective cohort study, we evaluated the incidence, clinical characteristics, antifungal susceptibility profile, and outcomes of *C. auris* bloodstream infection (BSI) compared with those of non-auris candidaemia at a large tertiary referral hospital in the Republic of Korea. During the 2-year study period, *C. auris* accounted for approximately one-tenth of all candidaemia cases. Despite a decline in overall candidaemia admissions in 2024, likely reflecting nationwide healthcare disruptions [26], the relative contribution of *C. auris* remained stable, indicating that *C. auris* has become a clinically important cause of candidaemia at our institution [27]. After inverse probability of treatment weighting (IPTW), *C. auris* BSI was not associated with higher adjusted mortality than non-auris candidaemia, and this null finding was directionally consistent across multiple sensitivity and alternative-method analyses; the precision of the estimates, however, was limited by the small number of *C. auris* cases.

Consistent with previous reports, patients with *C. auris* BSI were older, carried a heavier comorbidity burden, and were more frequently in the intensive care unit at diagnosis, in keeping with the close association between *C. auris* and prolonged or intensive healthcare exposure [12,13]. Differences in the distribution of individual comorbidities between groups most plausibly reflect distinct patient populations and pathways of healthcare contact rather than species-specific biological effects.

The absence of an adjusted mortality difference between *C. auris* and other *Candida* species is consistent with prior clinical reports [12,13] and with experimental data indicating that the intrinsic virulence of *C. auris* is generally lower than, or at most comparable to, that of *C. albicans* [17,19]. To our knowledge, this study is among the first to compare clinical outcomes of *C. auris* BSI with those of other *Candida* species in an East Asian population, where prior reports have largely focused on detection and infection control [23]. Because many patients in our cohort were managed in general wards rather than exclusively in intensive care units, our findings may better reflect real-world prognosis among hospitalised patients with candidaemia.

Several features of the comparison warrant emphasis. The comparator group was inherently heterogeneous, comprising multiple *Candida* species with differing virulence and susceptibility profiles; to address this, we compared *C. auris* separately against *C. albicans* alone and against non-albicans species, and the estimates remained directionally consistent. Concomitant bacteraemia, which could dilute a species-specific effect, and restriction to echinocandin-treated patients were likewise examined as alternative cohort definitions, again without altering the qualitative finding. Because the *C. auris* arm was small, these comparisons are best interpreted as showing that the null finding was not overturned by alternative analytic choices rather than as a formal demonstration of robustness. The temporal pattern of mortality also differed between groups: among patients who died, *C. auris* deaths were distributed later and were less concentrated in the acute post-onset period than comparator deaths. Because only a minority of patients in either group remained under observation through 90 days, we pre-specified 30-day mortality as the primary outcome, focusing the principal comparison on the early period in which most events occurred and follow-up was more complete, while also reporting 90-day, in-hospital, and day-14 mortality; we caution against strong interpretation of the later (90-day) mortality differences given the limited long-term follow-up.

Beyond the primary mortality comparison, our exploratory analyses of antifungal susceptibility and agent selection provide additional clinical context. *C. auris* isolates were almost uniformly fluconazole non-susceptible, with high-level caspofungin resistance in the large majority of tested isolates, while micafungin and anidulafungin susceptibility was preserved in most. This profile mirrors molecular epidemiology data from the Republic of Korea, where surveillance studies have reported circulation of clade I and clade II isolates with predominantly fluconazole-resistant, variably echinocandin-susceptible patterns, and where clade IV has also been documented in nosocomial outbreaks [21,22]. The species-level distribution further showed that caspofungin non-susceptibility in this cohort was concentrated almost entirely in two species, *C. auris* and *C. glabrata*, whereas *C. albicans*, *C. parapsilosis*, and *C. tropicalis* were nearly uniformly susceptible. This caspofungin-restricted pattern—non-susceptibility to caspofungin with retained micafungin and anidulafungin susceptibility—most plausibly reflects the well-recognised interlaboratory variability of caspofungin minimum inhibitory concentration testing rather than true echinocandin-class resistance; both the Clinical and Laboratory Standards Institute (CLSI) and the European Committee on Antimicrobial Susceptibility Testing (EUCAST) advise against relying on caspofungin for class-level susceptibility determination, and EUCAST has not established caspofungin clinical breakpoints for this reason [28,29,30]. Genuine echinocandin resistance is mediated by FKS1/FKS2 hot-spot mutations and confers cross-resistance across the entire class, so the preserved micafungin and anidulafungin susceptibility argues against FKS-mediated resistance in these isolates [31,32]. The paradoxical (Eagle) growth of *Candida* at supra-inhibitory echinocandin concentrations is a distinct in vitro phenomenon and does not account for this pattern [33]. Because isolates were not retested by a reference method and FKS genotyping was not performed, we interpret the caspofungin results conservatively and base echinocandin-class susceptibility on the micafungin and anidulafungin categories. Among echinocandin-treated patients, the choice between agents appeared to reflect both species-specific susceptibility and the baseline hepatic profile of recipients: patients selected for anidulafungin had markedly higher baseline bilirubin and a higher prevalence of liver disease than caspofungin recipients, consistent with the predominantly non-hepatic clearance of anidulafungin. We could not assess *C. auris* clade affiliation directly because genotyping was not performed within the cohort, and longitudinal liver-enzyme trajectories during echinocandin exposure were not consistently captured; both are addressed as limitations below.

Some limitations should be acknowledged. First, the number of patients with *C. auris* BSI was small, which limited statistical power and produced wide confidence intervals compatible with both clinically meaningful harm and clinically meaningful benefit; the findings should therefore be regarded as hypothesis-generating rather than as confirmation of equivalence. Second, although IPTW improved covariate balance overall, some residual imbalance persisted after weighting, given the limited propensity-score overlap between the small *C. auris* group and comparators (reported in Section 3) [34]. Doubly robust models, pre-specified to combine weighting with outcome-model adjustment, yielded directionally consistent estimates but do not eliminate this residual imbalance. Third, although microbiological clearance is reported for the full cohort, the time to clearance could be quantified only in the subset with serial follow-up cultures, and clearance was therefore reported descriptively rather than modelled. Fourth, the analytic cohort, although inclusive of all systemically treated patients regardless of first-line agent, still excluded patients who received no antifungal therapy and those who died before treatment, so the findings generalise primarily to patients who survived to receive treatment. Fifth, this was a single-centre study without molecular genotyping, which limits generalisability and precludes clade-level inference. Sixth, residual confounding by unmeasured factors cannot be excluded despite IPTW adjustment. Finally, source attribution relied on operational microbiological and clinical criteria and is inherently uncertain; for this reason, source was not used in the adjusted analysis, and including it as an additional propensity-score covariate did not materially change the estimate. In a small number of patients no antifungal was administered after the index blood culture—possibly reflecting culture contamination or sampling near the end of life—so the total duration of antifungal therapy is reported only for those with an ascertainable post-index treatment course. Similarly, antifungal susceptibility testing was performed at the discretion of the treating clinician rather than on every isolate, so the tested subset may not be fully representative of the cohort and the susceptibility findings should be interpreted as descriptive rather than as population-based estimates of efficacy.

## 5. Conclusions

*C. auris* has become an established cause of candidaemia in this East Asian cohort. After adjustment for measured confounders, we found no evidence that *C. auris* bloodstream infection carried higher mortality than candidaemia caused by other *Candida* species; the small number of *C. auris* cases nevertheless limited precision, and the confidence intervals remained compatible with both benefit and harm. The clinical importance of *C. auris* may therefore relate more to its persistence in healthcare environments and capacity for nosocomial transmission than to enhanced intrinsic virulence, underscoring the importance of continued surveillance, molecular characterisation, and infection control. Larger multicentre studies incorporating clade-level genotyping and antifungal susceptibility surveillance are warranted to characterise the clinical impact of *C. auris* candidaemia more precisely.

## Figures and Tables

**Table 1:** Baseline characteristics of the main cohort by *Candida* species. *C. auris* (n = 29) versus other *Candida* species (n = 394), 2023–2024. Continuous variables: median (IQR); categorical: n (%). *p*-values from Mann–Whitney U test (continuous) and Fisher’s exact test (categorical).

Characteristic	Overall N = 423 ^1^	*C. auris* N = 29 ^1^	Other *Candida* Species N = 394 ^1^	*p*-Value ^2^
Age (years)	68 (59, 75)	72 (68, 78)	67 (59, 75)	0.012
Sex				0.3
Male	238 (56%)	19 (66%)	219 (56%)	
Female	185 (44%)	10 (34%)	175 (44%)	
Body mass index (kg/m^2^)	21.9 (19.3, 24.6)	22.6 (20.8, 26.5)	21.8 (19.2, 24.4)	0.088
Hospital days before candidaemia	22 (9, 47)	45 (14, 60)	22 (9, 44)	0.042
Hospital stay ≥ 48 h before candidaemia	400 (95%)	27 (93%)	373 (95%)	0.7
Admission within prior 90 days	221 (52%)	14 (48%)	207 (53%)	0.7
Intravenous antifungal within prior 90 days	420 (99%)	28 (97%)	392 (99%)	0.2
Surgery within prior 90 days	69 (16%)	5 (17%)	64 (16%)	0.8
MDR organism within prior 180 days	168 (40%)	13 (45%)	155 (39%)	0.6
Neutropenia (ANC < 500/µL)	28 (6.6%)	1 (3.4%)	27 (6.9%)	0.7
Charlson Comorbidity Index	6.00 (4.00, 8.00)	8.00 (6.00, 10.00)	6.00 (4.00, 8.00)	<0.001
Kidney transplantation	11 (2.6%)	0 (0%)	11 (2.8%)	>0.9
ICU at antifungal initiation	80 (19%)	13 (45%)	67 (17%)	<0.001
Septic shock	24 (5.7%)	3 (10%)	21 (5.3%)	0.2

^1^ Median (Q1, Q3); n (%). ^2^ Wilcoxon rank sum test; Fisher’s exact test. Other *Candida* species comprised *C. albicans* (n = 144), *C. tropicalis* (n = 100), *C. glabrata* (n = 83), *C. parapsilosis* (n = 43), and other species (n = 24). Abbreviations: CCI, Charlson Comorbidity Index; ICU, intensive care unit; *C. auris*, *Candida auris*.

**Table 2:** Infection source and antifungal treatment characteristics of the main cohort by *Candida* species. Categorical variables: n (%). *p*-values from Fisher’s exact test. Concomitant Gram-negative infection (a propensity-score covariate) is defined objectively from culture data within 14 days of the index candidaemia. The per-pathogen breakdown of concurrent multidrug-resistant co-infection across the full main cohort is provided in Supplementary Table S3.

Characteristic	Overall N = 423 ^1^	*C. auris* N = 29 ^1^	Other *Candida* Species N = 394 ^1^	*p*-Value ^2^
Presumed source of candidaemia				0.2
Intravascular device	206 (49%)	10 (34%)	196 (50%)	
Urinary	64 (15%)	4 (14%)	60 (15%)	
Intra-abdominal	8 (1.9%)	1 (3.4%)	7 (1.8%)	
Unknown	145 (34%)	14 (48%)	131 (33%)	
Concomitant Gram-negative infection	189 (45%)	15 (52%)	174 (44%)	0.4
Concomitant bacterial infection	251 (59%)	13 (45%)	238 (60%)	0.12
First-line echinocandin	228 (54%)	17 (59%)	211 (54%)	0.7
First antifungal agent				0.8
Fluconazole	195 (46%)	12 (41%)	183 (46%)	
Caspofungin	167 (39%)	12 (41%)	155 (39%)	
Anidulafungin	38 (9.0%)	4 (14%)	34 (8.6%)	
Micafungin	23 (5.4%)	1 (3.4%)	22 (5.6%)	
Total antifungal therapy (days)	14 (7, 19)	13 (6, 16)	14 (7, 19)	0.4
Documented microbiological clearance	357 (84%)	26 (90%)	331 (84%)	0.6

^1^ n (%); Median (Q1, Q3). ^2^ Fisher’s exact test; Wilcoxon rank sum test. Presumed source of candidaemia was adjudicated from the electronic medical record (intravascular device, a concordant catheter-tip culture; urinary, a concordant urine culture with urinary tract infection features; intra-abdominal, a concordant intra-abdominal specimen). It is shown for description only and was not included in the propensity-score model. Abbreviations: *C. auris*, *Candida auris*.

**Table 3:** Clinical outcomes of the main cohort by *Candida* species. Crude mortality and IPTW-adjusted estimates. Primary outcome: 30-day all-cause mortality, reported as an IPTW-adjusted hazard ratio alongside a doubly robust estimate. Secondary outcomes: 90-day mortality (weighted Cox aHR) and in-hospital mortality (weighted logistic aOR). All estimates with 95% CI. The time-to-event outcomes (30- and 90-day mortality) account for right-censoring of patients lost to follow-up before the respective horizon; the hazard ratios and Kaplan–Meier estimates therefore reflect the at-risk population over time rather than a fixed proportion of the full cohort.

Outcome	*C. auris* (n = 29)	Other *Candida* (n = 394)	IPTW-Adjusted (95% CI)	Doubly Robust (95% CI)
30-day mortality (primary)	9/29 (31%)	138/394 (35%)	aHR 0.59 (0.26–1.32)	aHR 0.64 (0.28–1.49)
90-day mortality (secondary)	14/29 (48%)	177/394 (45%)	aHR 0.76 (0.43–1.35)	aHR 0.82 (0.47–1.42)
In-hospital mortality	15/29 (52%)	176/394 (45%)	aOR 1.01 (0.42–2.45)	—

The propensity score comprised five covariates: age, Charlson Comorbidity Index, septic shock, ICU admission at antifungal initiation, and concomitant Gram-negative infection. In-hospital mortality is a secondary, horizon-free outcome estimated by weighted logistic regression (aOR) and is not directly comparable to the time-to-event hazard ratios. The 90-day deaths (n = 191) coincidentally equalled in-hospital deaths (n = 191): 18 deaths occurring after discharge but within 90 days offset 18 in-hospital deaths occurring after day 90. Abbreviations: aHR, adjusted hazard ratio; aOR, adjusted odds ratio; CI, confidence interval; IPTW, inverse probability of treatment weighting.

**Table 4:** Pre-specified sensitivity analyses for the primary outcome (30-day all-cause mortality). Robustness of the primary IPTW-adjusted estimate to (Block ①) the definition of the patient set and (Block ②) the choice of analytic estimator. Block ① re-estimates the species effect in alternative cohorts under the same five-covariate propensity-score model; Block ② applies alternative estimators to the same main cohort. All estimates with 95% CI.

Block	Analysis	N **(***C. auris*)	Estimate (95% CI)
Reference	Main analysis—IPTW Cox (5-covariate PS)	423 (29)	aHR 0.59 (0.26–1.32)
① Alternative cohort	(i) Echinocandin-restricted cohort (n = 237)	237 (23)	aHR 0.42 (0.16–1.15)
	(ii) Bacteraemia-excluded	295 (18)	aHR 0.47 (0.13–1.71)
	(iii) Comparator = *C. albicans* only	173 (29)	aHR 0.66 (0.29–1.51)
	(iv) Comparator = non-albicans only	279 (29)	aHR 0.55 (0.24–1.26)
**②** Alternative method	Doubly robust IPTW	423 (29)	aHR 0.64 (0.28–1.49)
	Overlap weights (ATO)	423 (29)	aHR 0.92 (0.45–1.86)
	Landmark, alive at 48 h	397 (—)	aHR 0.64 (0.27–1.52)
	Firth penalised propensity score	423 (29)	aHR 0.58 (0.26–1.30)
	Fine–Gray competing risk (in-hospital)	423 (29)	sHR 0.93 (0.53–1.64)
	Source-adjusted IPTW (6-covariate PS)	423 (29)	aHR 0.65 (0.29–1.46)

Concomitant bacteraemia: Any positive non-*Candida* blood culture within 14 days of the index *Candida* BSI. The echinocandin-restricted estimate is re-fitted with the five-covariate model and therefore differs slightly from the originally submitted six-covariate estimate. Overlap weighting (ATO) targets the equipoise population and achieves near-exact covariate balance; the Fine–Gray model treats discharge alive as a competing risk for in-hospital mortality. Abbreviations: aHR, adjusted hazard ratio; sHR, subdistribution hazard ratio; ATO, average treatment effect in the overlap population; CI, confidence interval; IPTW, inverse probability of treatment weighting.

## Data Availability

The data that support the findings of this study are available from the corresponding author upon reasonable request.

## Supplementary Materials

The following supporting information can be downloaded at: https://www.mdpi.com/article/10.3390/jof12070495/s1, Table S1: Quarterly incidence of candidaemia by *Candida* species; Table S2: Individual comorbidity components by *Candida* species; Table S3: Per-pathogen concurrent multidrug-resistant bacterial co-infection by *Candida* species; Table S4: Antifungal susceptibility profiles by *Candida* species; Figure S1: Propensity score distribution by treatment group; Figure S2: Crude Kaplan–Meier survival curves.
