# Supplementary material for: Clinical Outcomes of Candida auris Versus Other Candida Species Bloodstream Infections: An IPTW-Adjusted Cohort Study in South Korea
Source: J Fungi (Basel). 2026 Jul 6;12(7):495. doi: 10.3390/jof12070495 (PMC13412397; doi:10.3390/jof12070495)

Supplementary Materials

Clinical Outcomes of *Candida auris* versus Other *Candida* Species Bloodstream Infections: An IPTW-Adjusted Cohort Study in South Korea

2026-06-23

Supplementary Materials for: Clinical Outcomes of *Candida auris* versus Other *Candida* Species Bloodstream Infections: An IPTW-Adjusted Cohort Study in South Korea

Supplementary Table S1. Quarterly incidence of candidaemia stratified by *Candida* species (2023–2024). Number of patients diagnosed with candidaemia per calendar quarter, stratified by *C. albicans*, *C. auris*, *C. parapsilosis*, *C. glabrata*, and other *Candida* species (all remaining species, including *C. tropicalis*, combined). Data are absolute counts per quarter for the 423-patient main cohort.

Quarter	C. albicans	C. auris	C. parapsilosis	C. glabrata	Others
2023-Q1	27	2	7	17	14
2023-Q2	19	2	10	12	17
2023-Q3	32	5	9	19	29
2023-Q4	14	8	12	13	14
2024-Q1	17	4	4	8	13
2024-Q2	11	1	2	3	4
2024-Q3	11	3	3	8	12
2024-Q4	13	4	1	3	16
Total	144	29	48	83	119

Supplementary Table S2. Individual comorbidity components by *Candida* species. The individual comorbidities comprising the Charlson Comorbidity Index (collapsed into the single index value shown in Table 1), presented by species for the 423-patient main cohort. The Charlson Comorbidity Index total is shown as median (interquartile range); individual components as n (%). *p*-values from the Mann–Whitney U test (continuous) and Fisher's exact test (categorical).

**Characteristic**	**C. auris N = 29**[1]	**Other Candida species N = 394**[1]	**p-value**[2]
**Charlson Comorbidity Index, median (IQR)**	8.00 (6.00–10.00)	6.00 (4.00–8.00)	<0.001
**Hypertension**	23 (79%)	247 (63%)	0.072
**Diabetes mellitus**	21 (72%)	191 (48%)	0.013
**Myocardial infarction**	12 (41%)	69 (18%)	0.002
**Cerebrovascular disease**	6 (21%)	42 (11%)	0.12
**Congestive heart failure**	17 (59%)	133 (34%)	0.007
**Peripheral vascular disease**	4 (14%)	34 (8.6%)	0.3
**Dementia**	8 (28%)	37 (9.4%)	0.007
**Chronic pulmonary disease**	7 (24%)	51 (13%)	0.10
**Connective tissue disease**	1 (3.4%)	24 (6.1%)	>0.9
**Peptic ulcer disease**	14 (48%)	92 (23%)	0.003
**Liver disease**	7 (24%)	65 (16%)	0.3
**Chronic kidney disease**	15 (52%)	116 (29%)	0.012
**Solid tumour / malignancy**	9 (31%)	254 (64%)	<0.001
**Leukaemia**	1 (3.4%)	7 (1.8%)	0.4
**Kidney transplantation**	0 (0%)	11 (2.8%)	>0.9

[1]Median (Q1–Q3); n (%)

[2]Wilcoxon rank sum test; Pearson's Chi-squared test; Fisher's exact test

Supplementary Table S3. Per-pathogen concurrent multidrug-resistant bacterial co-infection by *Candida* species. Concurrent multidrug-resistant (MDR) bacterial co-infection across the 423-patient main cohort, defined as an MDR organism from a blood culture, or from a respiratory specimen with concurrent pneumonia, within 7 days of the index candidaemia. Categories are extended-spectrum β-lactamase (ESBL)-producing Enterobacterales, methicillin-resistant *Staphylococcus aureus*, vancomycin-resistant *Enterococcus*, carbapenem-resistant Enterobacterales, multidrug-resistant *Pseudomonas aeruginosa*, and multidrug-resistant *Acinetobacter baumannii*; a patient may contribute to more than one. Values are n (%); *p*-values from Fisher's exact test. Distinct from prior MDR colonisation within 180 days (Table 1) and from surveillance stool cultures.

Characteristic	Overall N = 423[1]	C. auris N = 29[1]	Other Candida species N = 394[1]	p-value[2]
**Any concurrent MDR co-infection**	129 (30%)	9 (31%)	120 (30%)	>0.9
**ESBL-producing Enterobacterales**	35 (8.3%)	2 (6.9%)	33 (8.4%)	>0.9
**Methicillin-resistant Staphylococcus aureus**	5 (1.2%)	0 (0%)	5 (1.3%)	>0.9
**Vancomycin-resistant Enterococcus**	40 (9.5%)	3 (10%)	37 (9.4%)	0.7
**Carbapenem-resistant Enterobacterales**	51 (12%)	2 (6.9%)	49 (12%)	0.6
**Multidrug-resistant Pseudomonas aeruginosa**	23 (5.4%)	3 (10%)	20 (5.1%)	0.2
**Multidrug-resistant Acinetobacter baumannii**	28 (6.6%)	4 (14%)	24 (6.1%)	0.11

[1]n (%)

[2]Fisher's exact test

Supplementary Table S4. Antifungal susceptibility profiles by *Candida* species (blood isolates). Per-species count of susceptible / tested isolates with the proportion susceptible (S) and the count of resistant (R) isolates, on the index blood culture. Untested cohort patients (no clinical antifungal susceptibility testing result on record for that species) are shown in the row header. Per-patient worst-case result for each drug class. Susceptibility categories were taken as recorded in the electronic medical record.

Species	Fluconazole	Caspofungin	Micafungin	Anidulafungin
C. auris (n=29; 14 untested)	1/15 (7%) S; 14 R	2/15 (13%) S; 13 R	13/15 (87%) S; 2 R	12/13 (92%) S; 1 R
C. albicans (n=144; 69 untested)	73/75 (97%) S; 1 R	75/75 (100%) S; 0 R	75/75 (100%) S; 0 R	—
C. glabrata (n=83; 34 untested)	3/49 (6%) S; 11 R	26/49 (53%) S; 13 R	47/49 (96%) S; 2 R	—
C. parapsilosis (n=48; 25 untested)	20/23 (87%) S; 3 R	23/23 (100%) S; 0 R	22/23 (96%) S; 0 R	1/1 (100%) S; 0 R
C. tropicalis (n=100; 39 untested)	49/61 (80%) S; 12 R	59/61 (97%) S; 1 R	61/61 (100%) S; 0 R	1/1 (100%) S; 0 R
Other Candida spp. (n=19; 11 untested)	5/5 (100%) S; 0 R	8/8 (100%) S; 0 R	8/8 (100%) S; 0 R	—

Cells show: n susceptible / n tested (%S); n resistant. Per-patient worst-case result; blood isolates only.

S, susceptible; R, resistant; SDD, susceptible dose-dependent. "Untested" = cohort patients with no clinical AFST result on record for that drug.

Main cohort (N = 423); species identification by MALDI-TOF mass spectrometry, categories as reported by the clinical microbiology laboratory.

Supplementary Figure S1. Propensity Score Distribution by Treatment Group. Density plot of estimated propensity scores for patients with *C. auris* bloodstream infection and those with other *Candida* species candidaemia, calculated using the five-covariate propensity-score model. Overlap between the distributions indicates the region of common support for causal inference.

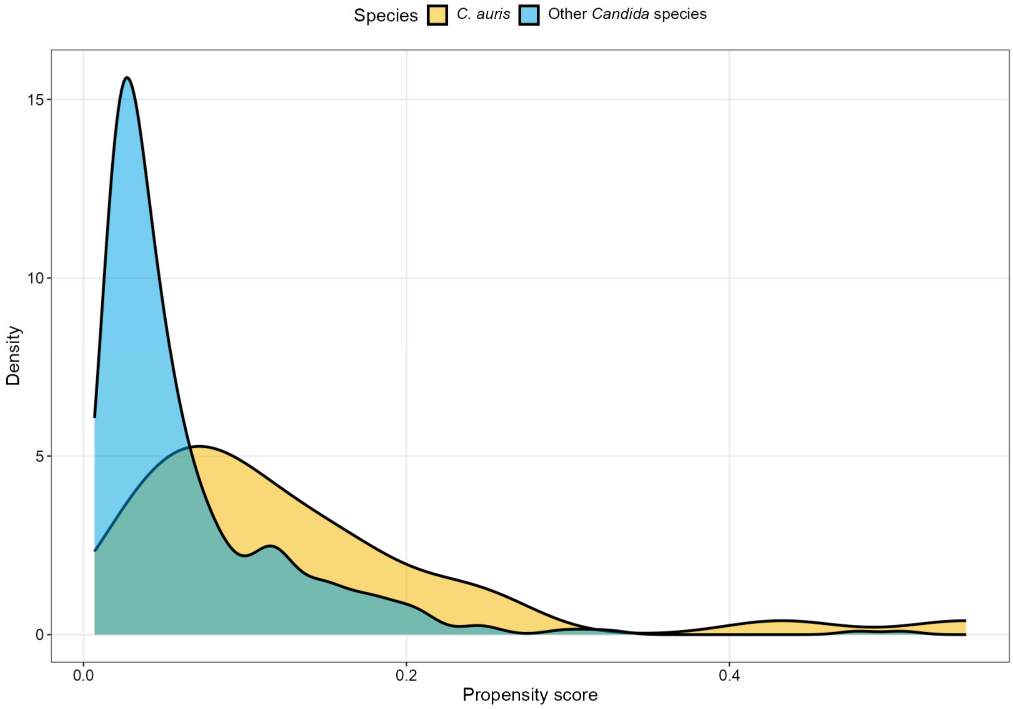

Supplementary Figure S2. Crude Kaplan–Meier Survival Curves. Unadjusted Kaplan–Meier survival curves for patients with *C. auris* bloodstream infection (n = 29) versus other *Candida* species (n = 394), with integrated numbers at risk through 90 days. Displays the crude (unweighted) mortality contrast; complements the IPTW-adjusted curves presented in Figure 4 of the main text.

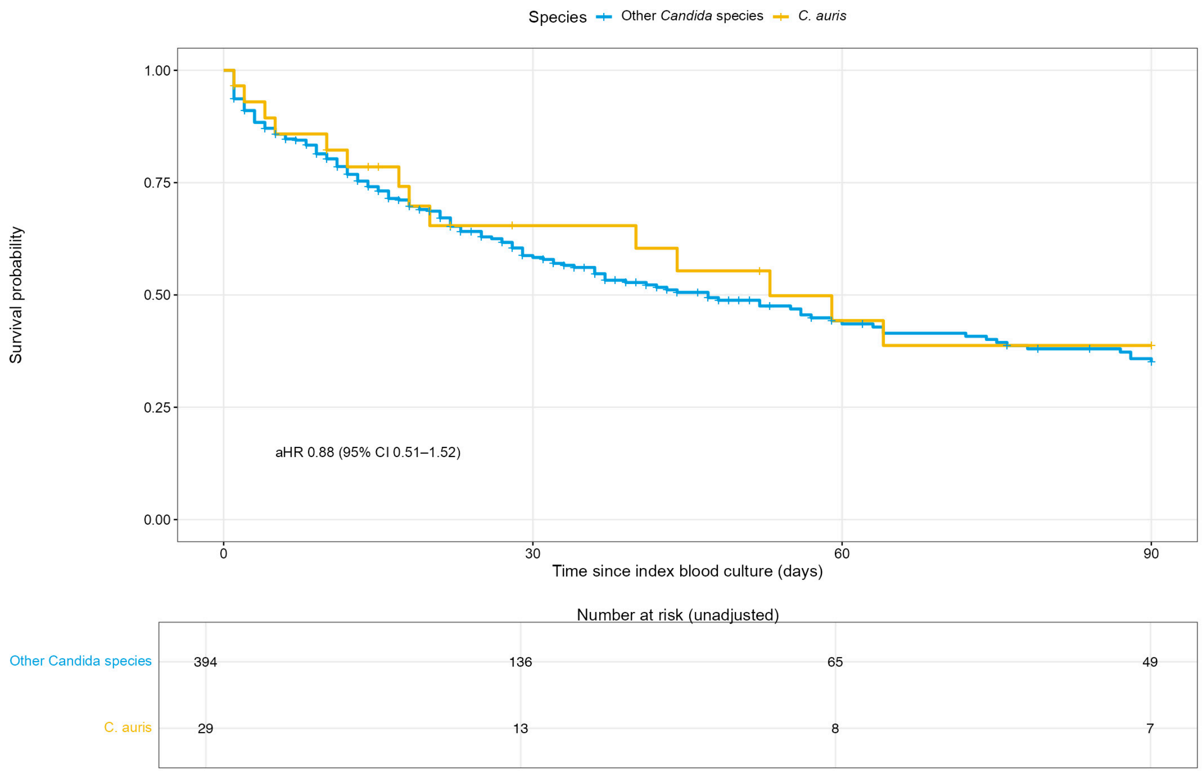

Supplement: Supplementary file 1 [file jof-12-00495-s001.zip › jof-4290046-supplementary.pdf]
